# Shock transmission in the International Food Trade Network

**DOI:** 10.1371/journal.pone.0200639

**Published:** 2018-08-08

**Authors:** Tiziano Distefano, Francesco Laio, Luca Ridolfi, Stefano Schiavo

**Affiliations:** 1 Politecnico di Torino - Dept. of Environment, Land and Infrastructure Engineering, Corso Duca degli Abruzzi 24, 10129 Torino, Italy; 2 University of Trento, Department of Economics and Management, Trento, Italy; 3 Observatoire Français des Conjonctures Économiques - Département de Recherche sur l’Innovation et la Concurrence (OFCE-DRIC), Sophia Antipolis, 60 Rue Fedor Dostoïevski, 06902 Valbonne, France; Pisa University, ITALY

## Abstract

Food Security is a long-standing concern worldwide. The expansion of global food markets brings benefits but also risks, such as shock transmission within the global network of trade relations. We focus on this last issue, from an empirical point of view, by analysing the diffusion of trade shocks—defined as relevant drops in exported quantities—during the period 1986—2011, for four major staples (wheat, maize, rice, and soy-beans) both at country level and at global scale. We find that: (i) income per capita of importing countries is relevant in shock propagation; (ii) developing countries tend to absorb most of the negative export variation (i.e., the trade shock), and (iii) global food prices and real (tonnes) flows of commodities are only weakly correlated, meaning that a quantity-based investigation provides additional information with respect to a price-based analysis. This work offers a novel framework, complementary to the price-based literature, for the measurement of the propagation of international food shocks.

## 1 Introduction

The production and provision of adequate food, in quantity and quality, is a long-standing concern worldwide (see [1, 2] for a detailed analysis of the evolution of the official definitions of food security). Food consumption has not grown linearly with population size due to the so called ‘Bennett’s law’, whereby the consumption of fats and proteins tends to increase with income per capita (e.g., [3]). The need to feed animals and the use of biofuels [4], are putting additional pressure on the food system and related water resource [5]. The recent spikes in food prices, occurred in 2007/2008 and in 2010/2011 (e.g., [6]), have pushed food security to the top of the global policy agenda.

From the 1980s onwards, international trade grew substantially, accounting nowadays for about 23% of the food produced for human consumption [7]. The last OECD-FAO’s Agricultural Outlook [8] underlines the importance of international trade to overcome local food supply shortages, although it might also expose importing countries to risks from external perturbations (e.g., [9–12]). Two main strands of literature attempt to face this issue: (i) economic models looking at food crises through the lens of price fluctuations, or (ii) simulations of ‘cascading shock’ models take the problem from a network science point of view. The two approaches are briefly discussed in what follows.

Economic analyses use food prices as indicators of the onset and propagation of food crises. However, they are affected by many other factors, beyond demand and supply, such as: GDP growth, monetary expansion, speculative finance, exchange rates [13] and energy [14, 15]. Additionally, price insulation [16], where (mostly developing) countries vary their protection to offset the effects of changes in world prices, is another important cause of changes in food prices. Several studies show complex effects of food price fluctuations, particularly in the developing countries [17].

The second strand focuses on the so called International Food Trade Network (henceforth IFTN, see [18]). Network analysis has been extensively used to analyse bilateral trade flows [19] and several indexes have been developed to assess the network vulnerability to exogenous perturbations (e.g., [20]). Recent applications regard the IFTN resilience: [21] focused on fish trade and identified Central Asia and West Africa as the most vulnerable region to external shocks, while [9] found evidence that local food-production crises propagate worldwide and that stronger effects of crises are expected in countries with low food availability, and [10] observed that trade and stocks make a country more resilient in case of national drop in production, but high import dependency increases the risk of food shortages following a foreign shock. Overall, both the linkage between increasing international trade and food security, and the identification of the most vulnerable areas, require further investigations.

We introduce a complementary approach to the existing literature. We follow the IFTN literature, but we focus on the actual consequences of past export shocks. The main research questions we address are: (a) How do local shocks transmit (in the short-term) to direct importers in the international food trade system? (b) What are the main drivers of shock propagation? (c) What is the effect of cross-country income inequalities? (d) What is the relation between export fluctuations and price dynamics? We answer to these questions by introducing an approach with the following features: (i) it is a data-driven study, (ii) it provides direct information on the potential lack of food, that can be hidden when looking only at prices, and (iii) it assesses the link between export-volume variations and price fluctuations in order to offer a wider framework to debate food security.

**Fig 1:**
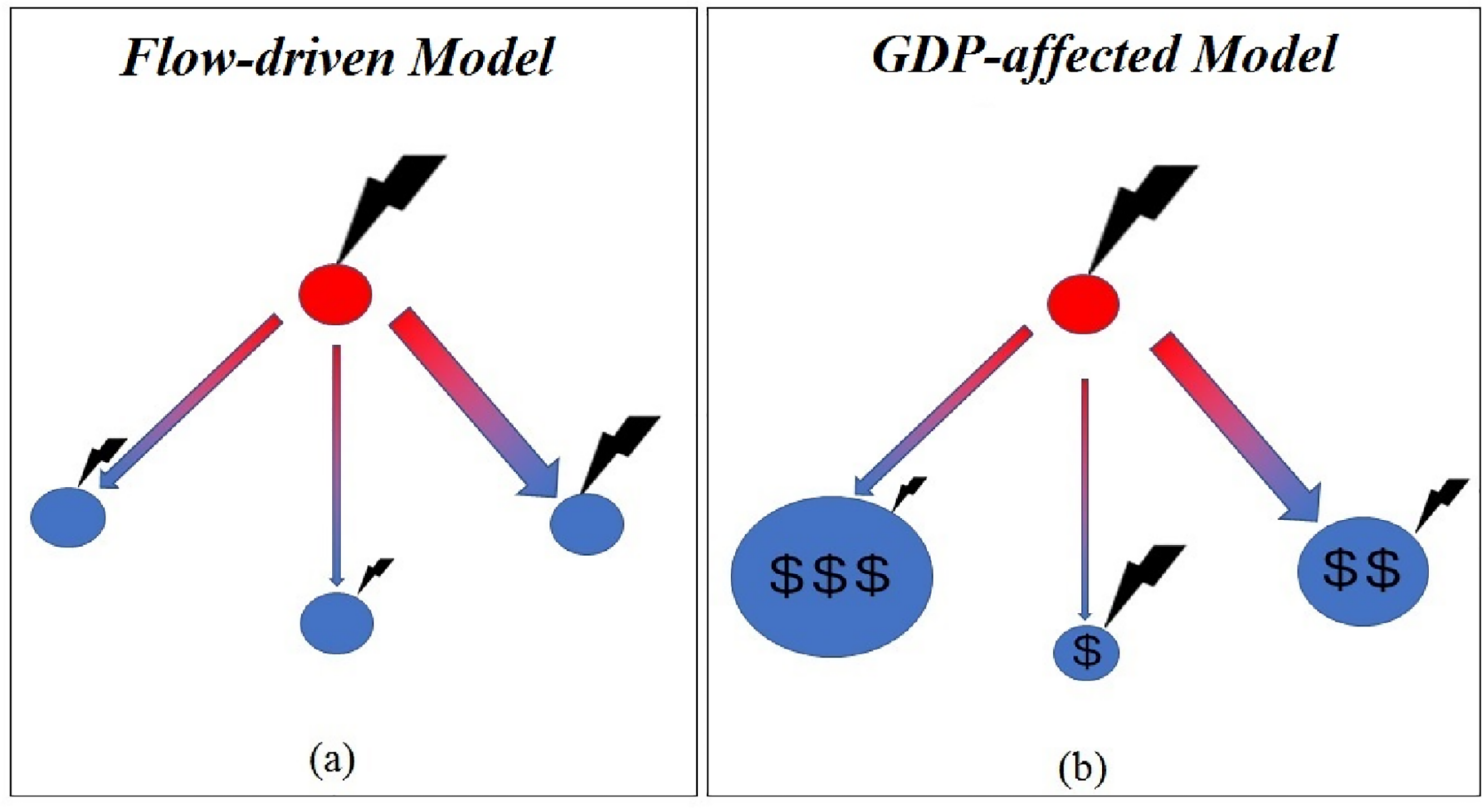
Two shock propagation models. Network representation of two trade shock (thunderbolt) propagation models. Panel (a) represents a simple “flow-driven” model whereby a negative shock (black thunderbolt) hits an exporter (red circle), then propagates in proportion to the (previous) level of bilateral trade flows (arrow size). Hence, each importer suffers from a reduction of food proportional to the share of import from the critical exporter and the shock higher for big importers (the dimension of the arrow is proportional to the tonnes exchanged). Panel (b) shows our “GDP-driven” model where the importers’ GDP per capita (dollar sign) alters the distribution of the shock. Note that the thunderbolt represents the reduction of bilateral flows toward all trading partners.

Fig 1 provides a graphical representation of our model, clarifying the aim and the novelty introduced in this work and showing how a shock propagates in the IFTN. Differently from previous studies (e.g., [10]), we allow economic variables (i.e., GDP per capita) to alter the distribution of the negative consequences of a negative shock. Our approach gives relevant policy indication for the introduction of safety nets for the more vulnerable countries.

The structure of the paper is as follows. Section 2 describes the database, Section 3 explains the methods and the econometric equation, and Section 4 presents the results of the regression. Section 5 focuses on global trade shocks, it makes a comparison between export fluctuations and price dynamics, and it discusses the main implications. Finally, Section 6 draws the main conclusions.

## 2 Data

Trade data are taken from the publicly-available Food and Agricultural Organization online database (FAOSTAT, [22]), which reports the yearly trade flows—both in tonnes and in monetary values—among countries for several commodities, from 1986 to 2011. Note that the original data contain some inconsistencies between the declaration of importers and exporters, so that aggregate values differ. To solve this problem, we apply an algorithm to pick the data from the most “reliable” countries (see [23] for a complete description of the procedure). Our database is the most complete and reliable database currently available, for agri-food commodities, both in terms of items (more than 300) and countries (255). The number of countries depends on the evolution of political boundaries over time. However, this fact does not alter the meaning of the results we obtained, because the set of countries is consistent in each year and a single country-pair represent an observation of a specific year.

FAOSTAT provides data on: Import (*M*), Export (*E*), bilateral trade (*F*), production (*Q*), global inflation rate (*d*) Gross Domestic Product (*GDP*), and Population (*POP*). In case of *M*, *E*, *Q*, and *F* there are both monetary (US dollar) and physical (tonnes) values. Note that FAOSTAT reports the exporter-reported values as f.o.b. (free on board), while importer-reported values as CIF (cost insurance and freight). However, this distinction does not affect our results since the algorithm is applied to the physical values (tonnes) and we recover the monetary values by multiplying them by the average exporting price. We use the World Bank Data on Gross National Income (GNI), and on the income threshold to distinguish between Low, Medium-Low, Upper-Medium, and High income countries. This study concentrates on four major crops (wheat, maize, rice milled equivalent, and soy-beans) because they cover more than 50% of the global calories intake. In particular, wheat ensures the 20%, rice the 16%, maize the 13%, and soy-beans the 8% of the global human calories intake [7].

The large time-window considered (28 years) and the high number of bilateral trade relationships considered (from ∼ 500 at the beginning of the period to ∼ 2000 at the end, for each commodity) allow us to run a robust estimation of the main determinants of shock propagation, with potential improvements in the understanding of food (in-) security. Each monetary amount is expressed in real US dollars in order to make them comparable over time and to get rid of inflationary effects. Given the presence of two partners, the exporter and the importer, the selection of the “right” deflator might be ambiguous. Indeed, if one opts, for instance, the Consumer (or Producer) Price Index of the exporter then all the values of the importers will be distorted, mostly in the case of two countries with different food baskets of consumption [24]. For this reason, we opt to select a single yearly global deflator to reduce possible distortions.

## 3 Methods

We define a “negative food–supply shock” (*NFSS*) any relevant (at least 10^6^ tonnes) country-level drop in food export occurred over a short period of time (less than five years). We focus on the first-round shock transmission impact, by looking at the consequences on the direct trading partners (importers) with no inclusion of indirect effects. Our choice is motivated by the fact that the system at hand is so complex that a clear assessment of second-round effects would be too complicated and results could be spurious. Since the IFTN is influenced by so many factors (economic, political, climatic, dietary, and so on), we rely on statistical inference, instead of defining absolute cause-effect relations. Note that, to the best of our knowledge, a unique and shared definition of “Food Crisis” (*FC*) is not available in the literature. Scholars usually identifies a FC as price spike and/or high price volatility, lead by the implicit assumption that prices always convey a proper signal of scarcity. However, we show that this assumption does not always hold and then, in order to avoid confusion, we opt for the term ‘shock’.

The current study aims at finding the building blocks for the future definition and modelling of trade shock propagation within networks. Indeed, most of the current studies (e.g., [10]) assume that, after a shock, any node (importer) reduces the volume of imports in proportion to its node degree (average number of links) or weighted degree (average volume associated to each link). We extend previous studies by including those variables, both endogenous and exogenous to the IFTN, which provide information about the way country-driven trade shocks propagate. For this reason, we perform an econometric analysis to find statistically significant relations, as described below. We define a *NFSS* as the difference between any sequence of local maxima and succeeding local minima in the time series of food exports by each country *j* (*E*_*j*_), such that:

$$\begin{array}{c} \Delta E_{j}\left(t_{0},\tau \right)=E_{j}\left(t_{1}\right)-E_{j}\left(t_{0}\right), \end{array}$$ (1)

where *τ* = *t*_1_–*t*_0_ (with *t*_1_ > *t*_0_) is the duration of the crisis, *t*_1_ is the year of the local minimum and *t*_0_ the year of the local maximum level of export from country *j* (viz. the starting year of the shock). Note that for the purpose of the current study, we only select cases in which Δ*E*_*j*_(*t*_0_, *τ*) < 0 that represent a *NFSS*. To clarify our procedure consider the following example: an exporter *j* shows the following sequence (e.g., from 2001 to 2007) of exports {100, 110, 80, 70, 90, 60, 75}. The series has two local maxima (*E*_*j*_(2002) = 110 and *E*_*j*_(2005) = 90) and two local minima (*E*_*j*_(2004) = 70 and *E*_*j*_(2006) = 60), so that country *j* suffers a drop of Δ*E*_*j*_(*t*_0_ = 2002, *τ* = 2) = 40 tonnes (110-70) during two years, and a reduction of Δ*E*_*j*_(*t*_0_ = 2005, *τ* = 1) = 30 tonnes (90-60) over one year.

Note that we do not investigate the causes of the drop in exports, but we take export variations as given and explore their short-term effects. Given the international market structure (see S1 File), we assume that the observed trade slumps are due to supply–side factors, rather than demand ones. Indeed, demand is typically highly fragmented and a single country is generally unable to influence global demand. Moreover, the time span of each observed trade shock (around 2 years), the increasing food demand, the long time needed for agricultural policy reforms, and the rigidity of dietary habits in the short-term, lead us to assess shock transmission from a supply-side point of view. Indeed, in the last decades there have been several cases of supply-driven export shocks due to lean production (for instance in Ukraine in 2002), export bans, as happened in case of rice in several Asian countries (e.g., India, Vietnam) in the aftermath of the financial crisis of 2008, or due to the climate conditions (as the drought in Australia, see [25]). A notable exception is demand for soya, where China plays a major role accounting for almost 70% of global demand in 2011 (see Fig B in S1 File). Interestingly, empirical results for soya differ from those relative to the other crops, thus corroborating our hypotheses.

For each country-level *NFSS*, we compute the ratio between the export decrease in the critical country *j* and the total (including *j*) variation in global exports (Δ*E*^*w*^(*τ*)), during the same time span, to verify the global impact of the local shock. The ratio reads

$$\begin{array}{c} \Theta_{j}\left(\tau \right)=-\frac{\Delta E_{j}\left(t_{0},\tau \right)}{\Delta E^{w}\left(t_{0},\tau \right)} \end{array}$$ (2)

Note that, by construction, Δ*E*_*j*_(*t*_0_, *τ*) < 0, while the denominator can take both positive and negative values. With this in mind, we distinguish three possible alternatives: if Θ_*j*_(*τ*) ≥ 0, other countries offset the loss in export from *j* (that is |Δ*E*^*w*^(*τ*)| > |Δ*E*_*j*_(*τ*)|); if -1< Θ_*j*_(*τ*) < 0, all (most) of the other suppliers have reduced their exports as well in the same time span; while if Θ_*j*_(*τ*) ≤ −1, the other countries have partially compensated the loss in export due to *j*, and therefore the main driver of global export reduction was country *j*.

When looking at the bilateral trade, we define the physical flow of commodities (in tonnes) from exporter *j* to importer *k* as *F*_*jk*_. We consider variables possibly affecting the impact of an export drop on each of the trade partners of country *j*. For this purpose, we estimate the following power-law function:

$$\begin{array}{c} \Delta F_{jk}\left(t_{0},\tau \right)∼\beta_{0}\cdot gdp_{k}{\left(t_{0}\right)}^{\beta_{1}}\cdot F_{jk}{\left(t_{0}\right)}^{\beta_{2}}\cdot {\left(-\Delta E_{j}\left(t_{0},\tau \right)\right)}^{\beta_{3}} \end{array}$$ (3)

where Δ*F*_*jk*_(*t*_0_, *τ*) = *F*_*jk*_(*t*_1_) − *F*_*jk*_(*t*_0_) is the import variation (either positive or negative) after the export trade shock, *gdp*_*k*_ is the GDP per capita of the importing country, and Δ*E*_*j*_(*t*_0_, *τ*) = *E*_*j*_(*t*_1_) − *E*_*j*_(*t*_0_) is the cumulative export variation from country *j* during the crisis. We expect that each driver affects the magnitude of Δ*F*_*jk*_(*t*_0_, *τ*) differently:

- *gdp*_*k*_(*t*_0_) is the pre–shock income per capita of importing countries and it is thought as a proxy of purchasing power. Note that this variable does not depend on the network structure, but is exogenous to the IFTN. The main idea is that rich countries can afford the detrimental effects of the crisis by paying a higher price and/or take advantage from a higher bargaining power. Indeed, basic foods have high-income elasticities in poor countries (and relatively high price elasticities), but become more income and price rigid as income rise [26]; hence, *β*_1_ should be negative.
- *F*_*jk*_(*t*_0_): it is the pre-crisis level of import and represents the trade dependency of *k* on *j*. It is crucial for two reasons: first, a big importer has higher variations in absolute terms (tonnes); second, it should have more bargaining power so that it could keep its pre–shock level of imports from *j*. Then, the sign of *β*_2_ is ambiguous: it is positive if the latter effect is smaller than the former, and *vice versa*;
–Δ*E*_*j*_(*t*_0_, *τ*) is included because a large trade shock is likely to have a larger impact on each bilateral trade flow; observed hence, we should observe *β*_3_ > 0;

Note that here we deal with tonnes variations, that can be either positive or negative (so that it is not possible to linearise the expression via a logarithmic transformation). For the sake of completeness, we also performed a log-linearised estimation on the relative variation, to say the ratio between the imports at the end (*F*_*jk*_(*t*_1_)) and at the beginning (*F*_*jk*_(*t*_0_)) of the crisis. In practice, we build an alternative model by substituting *F*_*jk*_(*t*_1_)/*F*_*jk*_(*t*_0_) to Δ*F*_*jk*_(*t*_0_, *τ*) as the dependent variable and then we linearise the expression through logarithm. The goodness-of-fit is captured by the adjusted coefficient of determination, computed as:

$$\begin{array}{c} R_{adj.}^{2}=1-\frac{SSE}{SST}\cdot \frac{n-1}{n-p} \end{array}$$ (4)

where *SSE* is the sum square of errors (from estimated values), *SST* is the total sum square (from the average) and *n* and *p* are the total number of observations and parameters, respectively. Note that to make the two different specifications comparable *R*^2^ is computed on absolute (tonnes) value. The poor performances of the log-linear model ($R_{adj.}^{2}<25\%$) lead us to keep the non-linear specification.

The non-linear least-squares estimation is run by pooling together data from the top ten crises ranked by magnitude (see Table 1), and looking at those importers with an import share of at least 0.5% from country *j* where the shock originates. The resulting sample covers more than 95% of the total export (for each commodity). Note that in each case we have one data point from any importer *k* that was a partner of exporter *j* independently of the duration of the shock; indeed, the left–hand–side is the cumulative difference of bilateral trade, and in the right–hand–side we have another difference (viz. the cumulative export variation) and two pre–shock (i.e. income per capita and total bilateral trade) variables.

**Table 1:** Top 10 country-level trade shocks for wheat, from 1986 to 2011. The table reports the volume (Δ*E*_*j*_(*τ*) in 10^6^ tonnes) and shares of reduction, the period of each crisis, the global variation (Δ*E*^*w*^), and the ranking of the critical exporter *j* before (*t*_0_) and after (*t*_1_) the shock. The index Θ_*j*_(*τ*) refers to Eq (2).

*Country*	Δ*E*_*j*_(*τ*)	$\frac{\Delta E_{j}\left(\tau \right)}{E_{j}\left(t_{0}\right)}$%	Years	Δ*E*^*w*^(*τ*)	Θ_*j*_(*τ*)	Ranking *t*_0_ → *t*_1_
*USA*	-14.9	-36.32	1988 - 1990	-12.7	-1.17	1 → 1
*Canada*	-11.7	-50.22	1987 - 1989	-6.3	-1.83	2 → 3
*USA*	-10.6	-33.02	2007 - 2009	21.6	0.49	1 → 1
*USA*	-8.9	-28.69	2004 - 2006	5.1	1.77	1 → 1
*Ukraine*	-8.8	-68.57	2009 - 2011	2.3	3.76	6 → 8
*Australia*	-8.5	-55.66	2006 - 2007	1.6	5.23	4 → 6
*Australia*	-8.4	-47.34	2000 - 2003	-5.2	-1.62	4 → 4
*Australia*	-7.3	-44.88	1986 - 1988	19.2	0.38	3 → 4
*Canada*	-7.1	-37.83	2000 - 2003	-5.2	-1.36	2 → 3
*Ukraine*	-6.9	-80.12	2002 - 2003	-11.1	-0.63	7 → 12

## 4 Results

We mainly focus on a specific commodity (wheat) for which we provide a detailed discussion of the results, while for the other crops we refer to the S1 and S2 Files. Table 1 reports the basic information on the 10 largest country-*NFSS* that hit the international trade of wheat from 1986 to 2011. The epicentres of these shocks were the biggest exporters, such as the USA, Canada, Ukraine and Australia. One might rebut this approach saying that a country–level analysis might hide the possibility of compensation from other countries. However, we show that our results are robust against this observation for two reasons: (i) rarely (less than 20% of the times) the other competitors were able to compensate (Θ > 0) the loss of food due to a country–level shock that then propagated worldwide, and (ii) the global analysis of Section 5 confirms that country-level results hold true even when we consider global *NFSS* (i.e., poorer countries are less resilient than richer ones).

The USA reported the most severe *NFSS*, with a peak value of around -15⋅10^6^ tonnes from 1988 to 1990. The relative reductions range from around -28% (USA) to around -80% (Ukraine, due to a lean year, see [27]). *NFSS*s have an average duration of around 2 years. Half the time the global variation of exports (during the same period of the country-level crisis) followed the same trend, meaning that a single big exporter can generate a worldwide lack of supply. Values of Θ_*j*_(*τ*) < -1 appear in four out of five cases, implying that in most of the cases other exporters have only partially offset the reduction due to country *j*. When looking at other products (see S2 File), one observes heterogeneous tendencies. The average duration of the shock is around 2-3 years. In case of maize and soya, the ten most severe crises are caused by three countries, of which the USA and Argentina are present in both the commodities. Rice shows a more diversified picture, with five (Asian) countries acting as epicentres of the crises. The different market structures are reflected in the values of Θ_*j*_(*τ*). Maize shows a negative Θ_*j*_(*τ*) value in only four cases, while soya and rice show an opposite proportion, probably due to lower international competition. In any case, only less then 20% of the times other exporters compensated the country–level *NFSS*.

Table 2 provides a cross–commodity summary of the estimated exponents of Eq (3). The sign of *β*_1_, associated with *gdp*, is always negative and statistically significant for three commodities with a value around -0.26 for wheat and about -0.6 for maize and rice. This confirms the findings of the literature (e.g., [26]) that income elasticities are lower in case of more affluent countries. In other words, a higher income per capita makes a country more resilient to negative trade shocks. Soya is the only exception where *β*_1_ > 0 but it is not statistically significant. To understand this anomaly it is crucial to interpret the result under the lens of market structure dynamics. From the 2000s onward, soya demand is dominated by China which increased imports by almost 30⋅10^6^ tonnes (as shown in S4 File). Since China is a country with a relatively low GDP per capita (around 3150$ in real terms, in 2011), but with a level of imports larger than any other country, then *β*_1_ is positive. These outcomes highlight that poor countries (with the exception of soya for the reasons explained above) suffer from higher negative import variations than rich countries in case of *NFSS*. This result concerns the distributive effect of uneven income (per capita) distribution worldwide It provides additional insights—to the current food price literature—about the relation between consumer budget constraints and food supply shocks, and about the divergence between developing and developed countries in (household) expenditure on food [28].

**Table 2:** Non-linear least square estimations. Estimations of the exponents of Eq (3), where * means significant at 90%, ** at 95% and *** at 99% level, with in brackets the standard error. $R_{adj}^{2}$ is the adjusted coefficient of determination, and Obs. is the number of the importers directly hit by the trade shocks.

Δ*F*_*jk*_	wheat	soya	maize	rice
Const.	-1317	-0.0492	-0.038	-0.014
*gdp*_*k*_(*t*_0_)	- 0.137*** (0.036)	0.178 (0.390)	-0.658*** (0.059)	- 0.232*** (0.046)
*F*_*jk*_(*t*_0_)	1.296*** (0.069)	1.012*** (0.07)	1.460*** (0.093)	1.111*** (0.058)
−Δ*E*_*j*_(*t*_0_, *τ*)	0.691*** (0.169)	1.389*** (0.300)	0.055 (0.217)	0.261** (0.127)
**$R_{adj.}^{2}$**	**0.72**	**0.60**	**0.75**	**0.79**
*Obs*.	266	164	191	261

For what concerns trade-related variables, the sign of *β*_2_ (corresponding to *F*_*jk*_(*t*_0_)) is always positive and significant, with values in the range 1.01-1.46. Hence, if a country has high volumes of pre-crises imports, then the variations in level should be higher as well. This entails that the bargaining power does not depend on the imported quantity, but rather on economic power (viz.*gdp*_*k*_(*t*_0_)). The coefficient *β*_3_—associated to overall *NFSS*—is, as expected, positive and significant meaning that a more severe shock has a bigger impact because the flows of goods are remarkably lower. The overall statistical significance of the estimations is high, with an (adjusted) *R*^2^ > 0.7 for each product, with the exception of soya for which it is 0.6 due to the different market structure (as explained in the S1 File). To summarize we find that:

1. in (almost) each case the exponent signs are significant and common to each product;
2. in the case of soy-beans the analysis of the market structure (*S*1) allows one to explain the seemingly inconsistent result;
3. a cross commodity comparison confirms that: *i)* poor countries suffer more from a *NFSS*, *ii)* the market structure (see details on the S1–S3 Files) is crucial to understand the possible diffusion (worldwide) of local crises, and *iii)* the entity of the *NFSS* is relevant as well (coefficients *β*_3_).

Note that: (i) results reported in Table 2 hold without any consideration of price fluctuations, during the period of trade shocks; (ii) the constant is negative because most of the import variations during the crises are negative by definition, (iii) the regressions are run on data coming from all the crises pooled together, therefore these relations are time-independent, This last outcome is further confirmed by the fact that our trade shocks are not concentrated in a specific time window, nor they show cycles, rather they hit the IFTN system without any regularity over the time span considered.

Finally, to check the robustness of these estimations and to avoid spurious results, we also performed the same regressions with other variables, such as population size, stock variations, price and geographical distance between the country of origin and destination, these were not significant and did not alter our results. In particular, there were no evidence of strategic stock variations by the importers, since they were utterly unrelated with import variations. Note that the data on food stocks might be affected by uncertainties associated with data sources, accounting protocols, and whether stocks are recorded at the beginning or at the end of the growing season (e.g., [29]). Further analysis, ideally on more accurate data, is therefore necessary before one could advance any definitive conclusions on the actual role played by stock variations, but this lies beyond the scope of the present paper.

## 5 Discussion from a global perspective

In this Section, we offer a wider framework in which analysing the overall effects of trade shocks and the relations between physical flows and price fluctuations. Since here we deal with global values, we sometimes use the term ‘crisis’ instead of trade shock because, in this Section, the global negative food–supply shock (*G*–*NFSS*) represents an absolute lack of food in the whole IFTN, while in the previous analysis a negative shock in a country might be compensated by other exporters (although, as shown in Table 1, this rarely occurred). Note that, due to data availability, we based our analysis on annual prices, as commonly done in the literature. However, since inter-annual volatility is higher than intra-annual volatility (e.g., [30]), and because our econometric estimation is based on real flows (tonnes), we would expect that the key outcomes hold even at lower temporal scale (i.e., higher frequency). We compare the global time series of exports with the global average commodity price per ton (*ρ*) computed as (neglecting the time specification):

$$\begin{array}{c} \rho =\sum_{j}^{N}\left(p_{j}\cdot m_{j}\right) \end{array}$$ (5)

where *p*_*j*_ is the average export price per ton of exporter *j*—calculated as the ratio between the total monetary real value and the aggregate physical volume (tonnes) of export of country *j* in each year—and *m*_*j*_ is the market share of country *j*. Note that the our definition of weighted average global price returns values very close to those provided by the USDA (see http://www.indexmundi.com/commodities/).

**Fig 2:**
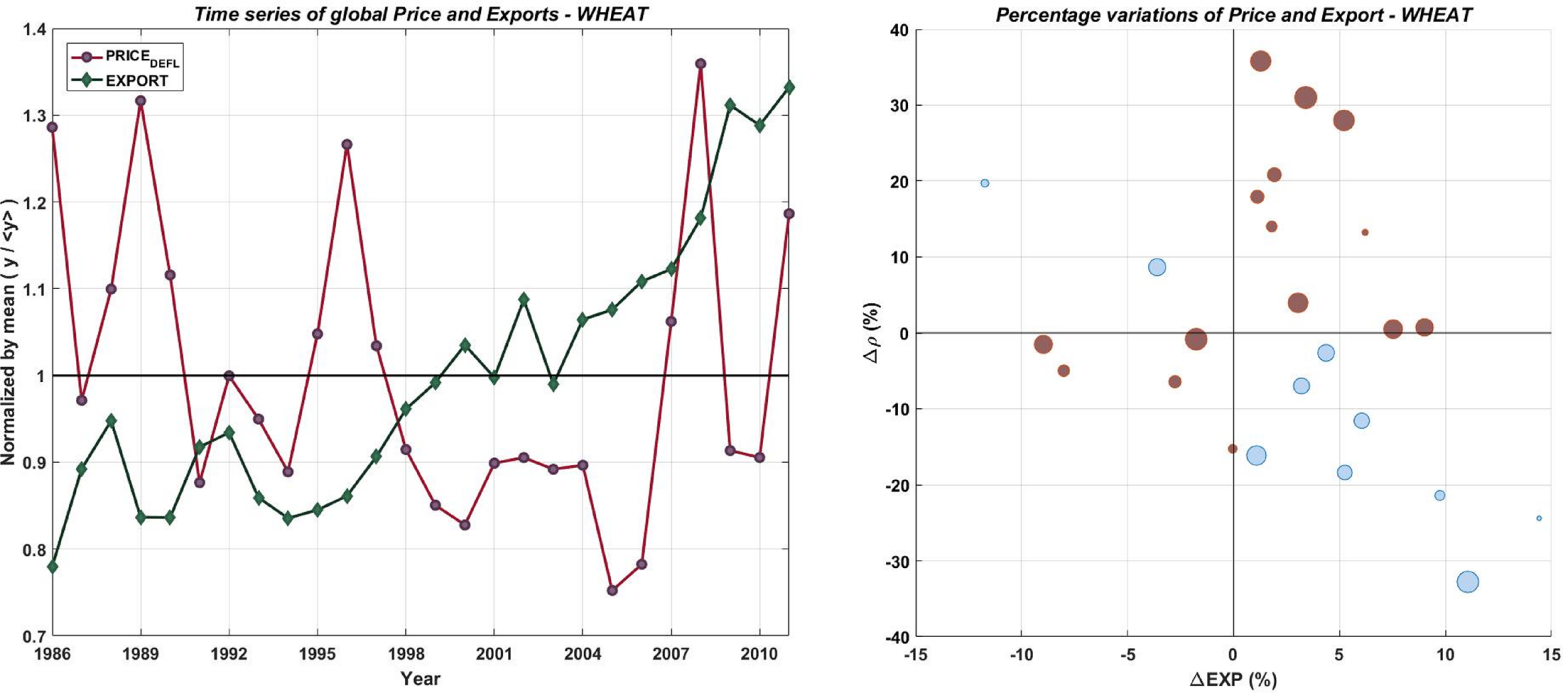
Global exports and prices over time. Left panel: time series of average global price and global export, normalised by mean, for wheat. Right panel: scatter plot of yearly percentage variations of global price and export. Red dots stand for positive correlation and blue dots for negative ones.

Fig 2 shows the time series of the wheat global price (*ρ*) and the yearly global export (*E*^*w*^), together with the scatter plot of the percentage variation of *ρ* and *E*^*w*^ computed with respect to the previous year. The left panel compares the dynamics of prices and exports, normalised by their weighted (by country market share) averages. From year 1994, *E*^*w*^ grew almost constantly, while prices followed an irregular path with several peaks and spikes. The two series are positively correlated (+0.69).

This observation challenges the assumption that price spikes always entail a reduction in the amount of food exchanged in the IFTN, with important implication for policy makers. Remarkably, during the two recent food price “crises”, in 2007/2008 (+33%) and 2010/2011 (+38%), *E*^*w*^ grew as well (of about +5.5% and +3.2%, respectively). The right panel shows that half of the times (13 out of 25, red dots) Δ*ρ*% and Δ*E*^*w*^% have the same sign, implying that in case of increasing (decreasing) supply the price grew (decreased) as well. The other commodities show similar results. In every cases the correlation coefficient between *ρ* and *E*^*w*^ was high and positive (from about +0.5 for rice to more than +0.7 for maize and soya). Based on these results, we think that the current food price literature might find useful information in the analysis of quantities exchanged, because food prices do not always properly signal scarcity in the global market.

We now evaluate the effects of global export drops, to verify if poor countries are more vulnerable and if global prices identify the crises properly. For each commodity we pick the five worst global trade shocks that, in general, correspond to a slump of at least 1 million tonnes in the global IFTN. We divide the importers hit by the crisis in four categories—depending on their Gross National Income (GNI) per capita per year (henceforth *gni*, in U.S. dollars, converted from local currency)—based on the World Bank (henceforth WB) classification: Low (*L*), Lower-Middle (*ML*), Upper-Middle (*UM*), and High (*H*). Each year these thresholds are updated, with an adjustment for inflation; on average (with a variation around ± 10%), they correspond to: Low if *gni* (real terms, deflated) < 840$, *ML* if *gni* < 3350 $, *UM* if *gni* < 10200 $, and High otherwise. To obtain these values, we compute the average of the deflated values of each threshold (real *gni*) from 1986 to 2011. Table 3 summarizes the main information on the distributional effects the five largest *G*-*NFSS*, and the price reactions in the same period.

**Table 3:** Income group comparison for the 5 worst global trade shocks of wheat in the period 1986-2011. Δ*F*_*k*_(*τ*) and Δ*E*^*w*^ are expressed in million of tonnes, while $\frac{\Delta F_{k}\left(\tau \right)}{POP}$ in kg per capita. Price are deflated and computed following Eq (5). The average percentage $\phi_{k}=\frac{\Delta F_{k}\left(\tau \right)}{F_{k}\left(t_{0}\right)}$ for each income group are in brackets. *The values corresponds to what we would obtain with the modified WB thresholds.

*Global Crisis*	Δ*F*_*k*_(*ϕ*_*k*_)				Δ*E*^*w*^	$\frac{\Delta F_{k}\left(\tau \right)}{POP}$				Price ($/ton)		
	*Low*	*ML*	*UM*	*High*		*Low*	*ML*	*UM*	*High*	$\rho_{t_{0}}$	$\rho_{t_{1}}$	Δ*ρ*(*τ*)
I. (1988-90)	-4.5 (-18%)	-10 (-24%)	0.1 (1%)	1.3 (6%)	*-13.1*	-1.60	-12.36	0.18	1.61	125	144	**+19**
II. (1992-94)	-4 (-15%)	3.7 (17%)	-16.5 (-56%)	5.4 (19%)	*-11.4*	-1.21	6.64	-24.96	5.98	142	123	**-19**
III.(2002-03)	-0.4 (-3%)	-4.8 (-12%)	2.2 (11%)	-7.9 (-16%)	*-11*	-0.19	-2.21	2.79	-8.20	125	140	**+15**
IV.(2000-01)	-1.7 (-9%)	-1.6 (-4%)	-3.3 (-16%)	2.2 (6%)	*-4.3*	-0.69	-0.79	-4.48	2.25	118	124	**+6**
V.(2009-10)	-1 (-7%)	-0.9 (-2%)	-1.3 (-3%)	0.6 (1%)	*-2.7*	-1.05	-0.38	-0.56	0.53	212	218	**+6**
**TOT**	*-11.6* (-10.3%)	*-13.6* (-4.8%)	*-18.8* (-12.6%)	*1.5* (3.0%)	**-42.5**	-4.75	-9.10	-27.03	2.18	-	-	-
TOT*	*-11.6* (-10.3%)	*-32.7* (-14.1%)	*+0.3* (+0.07%)	*1.5* (+3.0%)	**-42.5**	-4.75	-27.13	+0.52	2.18	-	-	-

During the five most severe global export dearths, the IFTN faced an overall drop of about -42.5 million tonnes of wheat, mostly concentrated in the top three crises (about -35.5 million of tonnes, almost the 85%). The average duration is around 2 years, similarly to the local crises seen above. In line with our previous findings, most of the loss of food is transmitted toward poor countries. *Low* and *ML* income groups, pooled together, faced a reduction of about -25⋅10^6^ tonnes (almost the 60% of the overall variation), that corresponds to about the 15% of their pre-crisis level of import, and to a loss of about -14 kg per capita of wheat. However, the picture is even worse if one looks at the income thresholds and into the crises in more detail. Indeed, the range of variation of the *UM* income cohort is quite large, where the maximum is about three times the minimum (i.e., [3350$, 10200$], on average), and we might imagine that the living standard of people who lives in a country closer to the lower bound (3350$) might be very different from that of people living in a richer country, with a *gni* close to 10000$. If we slightly shift the lower bound of *UM* to about 4500$, the condition of the poorer becomes far worse. In this case, the main loss of wheat is concentrated exclusively in the *Low* and *ML* income groups, while the other two classes face an increase in imports of almost 2 million tonnes, despite a global drop in exports. This suggests that most of the shock that hits the UM income group is actually concentrated around its lower bound, and behaviour within each income group is not homogeneous. Such finding is relevant when looking at riskier countries in case of trade shocks [31].

**Fig 3:**
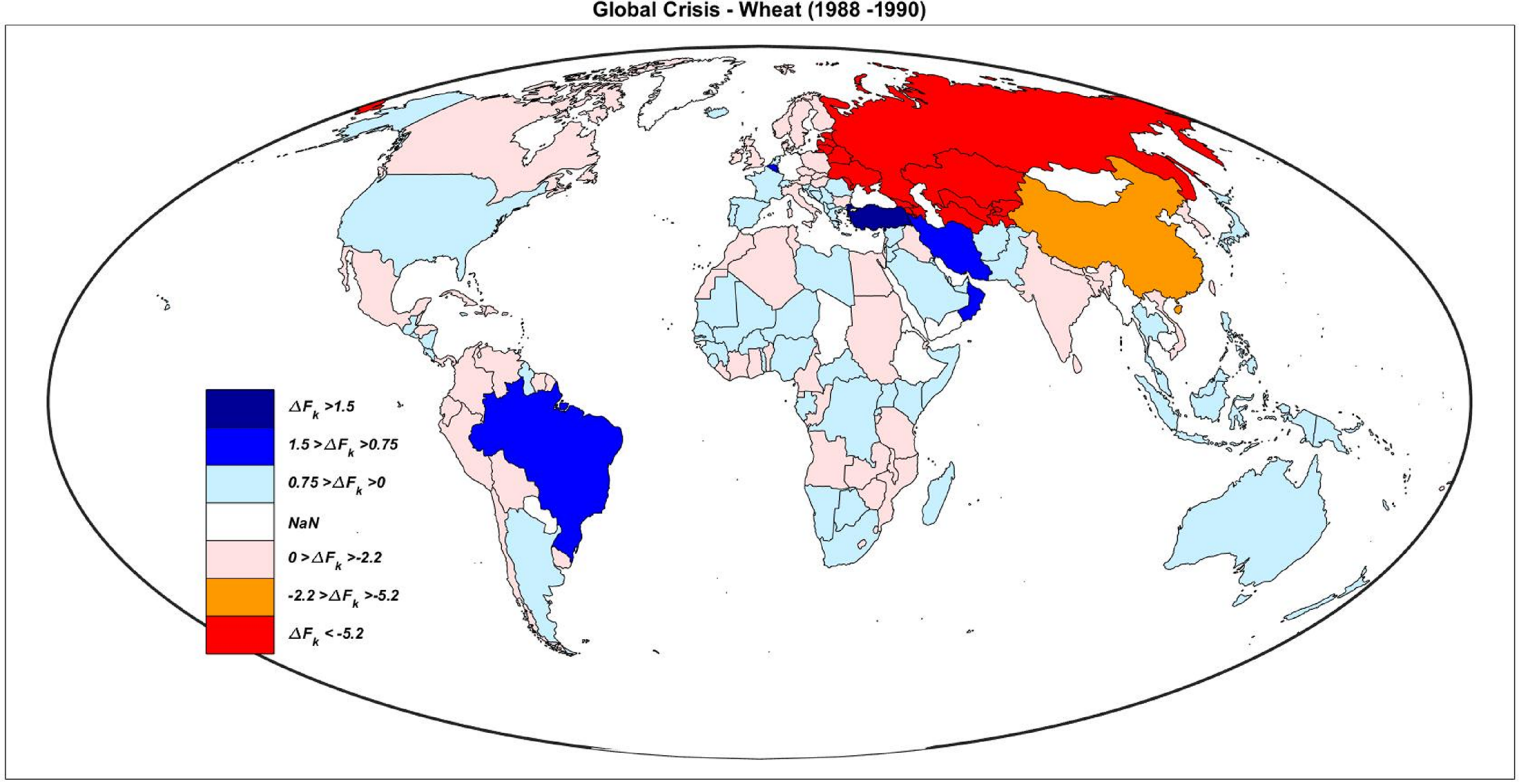
Global map of the effects of a G–NFSS. Effects of the worst global crisis of wheat (1988-1990, top), and the forth one (2000-2001, bottom). The negative import variations are in red (the darker the worse), while the positive ones are reported in blue (the darker the higher).

Note that global export drops include the overall effects, so that the results are even stronger than local crises, in which an importer could compensate by buying from different exporters. Fig 3 shows which countries increased (blue) or decreased (red) their imports during the worst global trade shock. In both cases (Fig 3A and 3B), we observe that Asian countries together with other Latin America and African countries (however Africa during the 1990s was scarcely participating to the IFTN) suffered more.

When looking at price variations we find results that further confirm the weak tie between export and prices, as reported above (see Fig 2). In two cases (Crisis IV and V) real global prices showed little variations of ∼+6$/ton although a reduction of more than 2 millions of tonnes was observed; while only in two cases prices significantly grew, of about 15 and 19$/ton. However, in the second worst negative trade shock—accounting for more than 11 million of wheat lost—the price variation was negative (-19$/ton). In any case, poorer countries almost absorbed the entire loss of food in the IFTN. The other commodities show results in line with these findings (see S3 File).

A question arises from this analysis: if prices do not always identify trade shocks, why are poor countries still the most vulnerable to (local and global) export slumps, even when prices are stable? We conjecture that a possible explanation might be found in the governance of the world trade and, more specifically, in the setting and evolution of International Agreements on Agriculture (IAoA). Recently, emphasis has been placed on how to set food policies in response to trade shocks [32, 33]. Starting from the General Agreement on Tariffs and Trade (GATT), signed in 1947, and moving through the Uruguay (spanning from 1986 to 1994), the Doha (2001) and the Nairobi (2015) WTO Rounds, an increasing number of countries have been involved in the definition of international rules on food trade, government subsidies, and tariffs. The broad trend are toward an increasing liberalization of trade (market-oriented), the elimination of export subsidies, the stabilisation of prices, and the harmonisation of agricultural policies. Many scholars and ONGs claim that historical multilateral IAoA have distributed commercial opportunities disproportionately to the largest players (e.g., [34, 35]). Several studies have tried to evaluate the effect of IAoAs on developing countries, with mixed results [36]. However, the assessment of the impact of the IAoA in the shock propagation is beyond the scope of this paper, but it represents an interesting extension for future researchers.

## 6 Conclusion

Food Security is a complex matter both to evaluate and to manage. The increasing relevance of global trade as a channel to meet rising food demand raises the question of how international *NFSS* transmit. Our study offers a novel view of the issue at stake. There are some key messages that deserve attention when debating on food “crisis” and on the policy intervention:

- looking at real flows (tonnes) allows one to directly evaluate the lack of food in the IFTN. It emerges that trade shocks are more frequent and severe than expected if one would have looked only at price ‘spikes’ (sometimes providing a misleading information, for instance when the price decreased during the second world wheat crises, or by noticing that the worst physical (in tonnes) *NFSS* did not take place during the “food price crises” of 2008 and 2011). It entails that price variations alone sometimes fail to identify real *NFSS*;
- income per capita plays a key role in the mechanism of transmission of trade shocks. The fact that income per capita matters, even when prices do not change significantly, suggests that income levels may not only capture purchasing power but may also reflect bargaining power or market access;
- poor countries tend to suffer larger import drops in the aftermath of a negative trade shocks, either local or global. This feature has not changed over time. In case of rice, wheat, and maize *low* and *medium*−*low* income countries absorb most of the loss of food during the most severe crises. These circumstances call for an increasing effort toward the definition of a set of tools to ensure food security worldwide. Otherwise, developing countries might be doomed to serious risks of instability in the provision of food. Since our study suggests that poorer countries face larger falls in food imports, then future international agreements should not simply focus on food price volatility but they should explicitly take into account the resilience of poorer countries (that we showed to be lower even in absence of any price spike);
- prices are a useful but incomplete tool to detect negative trade shocks (both at global and country-level) and to evaluate their effects. Prices matter but cannot be the only way through which one assesses and manages risks. Our approach confirms previous findings about the possibility that food prices may not always send the right signals about scarcity [37]. Therefore, an analysis based on physical quantities is useful to provide a complementary framework about the distributional effects of a *NFSS*.

These points, in addition to suggesting new insights on the transmission of trade shocks, open new questions for future research. The integration of quantity-based analysis with price-based indicators, in the direction of a multi-dimensional approach that include access to food, caloric intake, food quality, and so forth, can provide us with additional insights to understand and measure food security. Second, cross-country income inequality may have serious consequences for developing countries. Hence, trade negotiations should take into account the vulnerability of poorer countries to trade shocks. The recent WTO Nairobi Round (2015) is an example along these lines, but additional efforts are needed.

## Supporting information

S1 File The market structure. (PDF)

S2 File Country-scale trade shocks. (PDF)

S3 File Global-scale trade shocks. (PDF)

S4 File Supplementary figures. See “S4_File.rar”.
(RAR)
