# Correction: Shock transmission in the International Food Trade Network

**DOI:** 10.1371/journal.pone.0254327

**Published:** 2021-07-06

**Authors:** Tiziano Distefano, Francesco Laio, Luca Ridolfi, Stefano Schiavo

Fig 3 is incorrect. The authors have provided a corrected version here.

**Fig 3:**
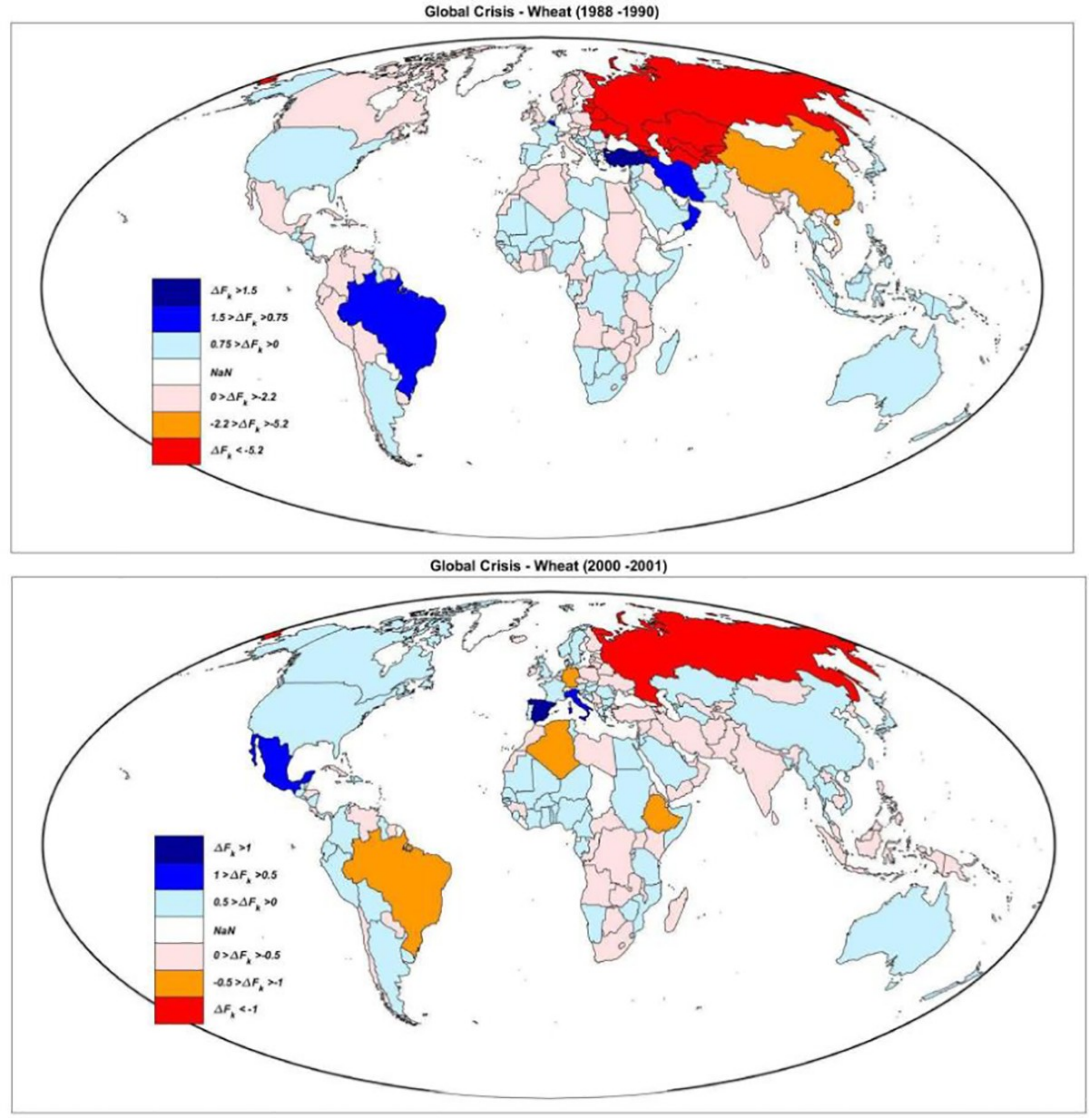
Global map of the effects of a G–NFSS. Effects of the worst global crisis of wheat (1988–1990, top), and the forth one (2000–2001, bottom). The negative import variations are in red (the darker the worse), while the positive ones are reported in blue (the darker the higher).
