# Supplementary material for: Shock transmission in the International Food Trade Network
Source: PLoS One. 2018 Aug 8;13(8):e0200639. doi: 10.1371/journal.pone.0200639 (PMC6082532; doi:10.1371/journal.pone.0200639)
Supplement: S1 File — (PDF) [file pone.0200639.s001.pdf]

Supporting Information

S1 File. The Market Structure

Export (E) represents an increasing portion of Production (Q) that goes from around 13% (maize) at the beginning of the investigated period (i.e., 1986), to more than 45% (soy-beans, henceforth soya) in 2011. Table 1 resumes the main information on production and exports, at the beginning (1986) and at the end (2011) of the time window considered.

Table A: Production and Export comparison. *Cross commodity comparison (between 1986 and 2011) of: the percentage of variations of production (Q) and exports (E), the ratio of exports to production ($S = E/Q$), the yearly growth rate (g) of Q and E, and the levels of Q and E, both in millions of tonnes (mmt) and in kg per capita. *Data on rice production in 2011 is recovered by Piesse and Thirtle (2009, pag. 128), based on their forecast for 2009.*

Item	Production			Export			Ratio	
	$\mathbf{Q}^{mmt}_{1986}$ (kg_{CAP})	$\mathbf{Q}^{mmt}_{2011}$ (kg_{CAP})	$\Delta Q\%$	$\mathbf{E}^{mmt}_{1986}$ (kg_{CAP})	$\mathbf{E}^{mmt}_{2011}$ (kg_{CAP})	$\Delta E\%$	$\mathbf{S}_{1986}$ ($\mathbf{g}_{Q\%}$)	$\mathbf{S}_{2011}$ ($\mathbf{g}_{E\%}$)
maize	406.2	694.3	+70.9	55.7	127.1	+128	13.7%	18.3%
	(83)	(99.7)		(11.4)	(18.3)		(2.65)	(3.68)
rice	-	450*	-	8.44	27	+219.9	-	-
		(64.6)		(1.7)	(3.9)		-	(5.94)
soya	94.4	261.6	+177.1	27.7	118.2	+326.7	29.3%	45.2%
	(19.3)	(37.6)		(5.6)	(17)		(4.39)	(6.55)
wheat	527.9	695.7	+31.8	89.4	152.7	+70.8	16.9%	21.9%
	(107.8)	(99.9)		(18)	(21.9)		(1.26)	(2.35)

The growth rate of E is always higher than that of Q for each product, meaning that a growing proportion of food production is traded. It is thus sensible to focus on the potential risks of the international food trade network. The share of E/Q is floating around 20% in 2011 for maize and wheat, while soya presents a much greater share, where almost half of Q is not consumed in the place of production but is traded worldwide. Soya experienced a striking growth in production, which is almost tripled, while the export increase was even faster, with a total variation of +328% in 26 years. The production of wheat is the most important, providing an average of almost 108 kg per capita worldwide (although it slightly declined in the last 26 years), of which almost 22 kg are exchanged in the international trade system. Timmer (2010) reports that only the 7-8% of rice produced crosses an international border, which is a relatively low share if compared with the other

staple foods, confirming that the international rice market is thin (Piesse and Thirtle, 2009), both in percentage and absolute terms.

Fig 1 shows the evolution of the market structure for wheat, with the volume of exports of the biggest importers and exporters from 1986 to 2011, compared with the Rest of the World (RoW). From the supply side, we observe a sharp decrease of the market share of the top 5 exporters, which passed from handing more than 80% of the market in 1986, to less than 70% in 2011, with a minimum of 60% touched in 2002 and 2009 (after the crisis).

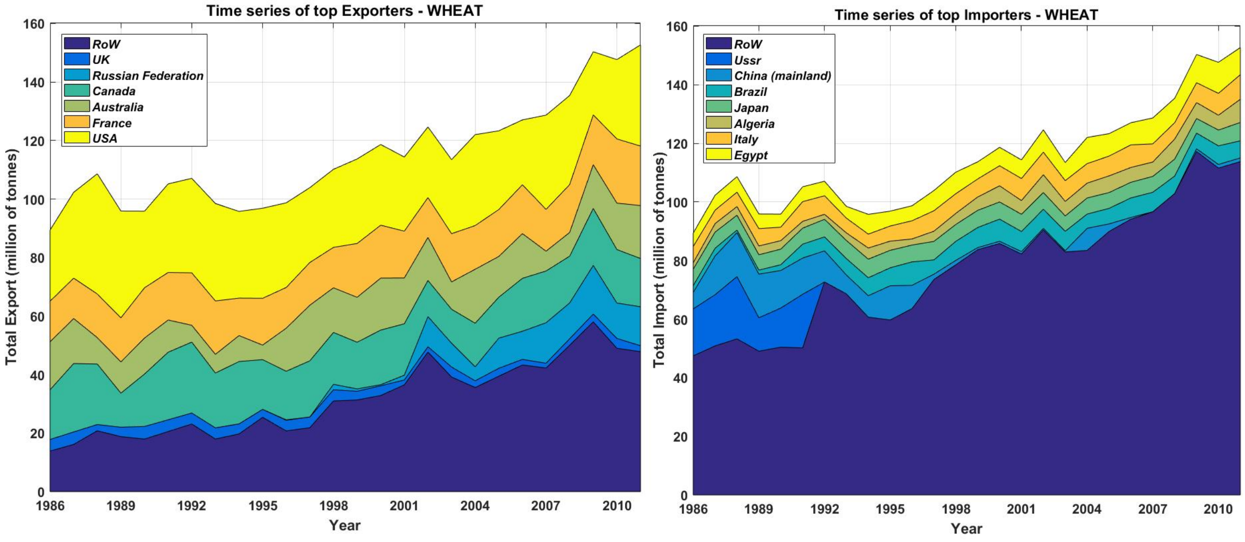

Fig A: Top exporters and importers over time (wheat). *Historical trend of the main export fluxes (left) and import fluxes (right), from 1986 to 2011, in million of tonnes. The Figure shows the time series of the exporters and importers which were among the top 5 in 1986 and/or in 2011.*

Fig A (left side) confirms the historical leadership of the USA which supplied, in 2011, about $35{\cdot}10^6$ tonnes (22.6%), followed by France (13.3%), Australia (11.8%), and Canada (10.8%) which was the second most important exporter in 1986 (accounting for the 20% of the market) but is losing share over time. Recently, the Russian Federation has become a relevant exporter, with a market share of 8.7%, due to the revision of its agricultural structure and policy, shifting from being the first importer worldwide in 1996 to becoming among the top exporters in 2011. Headey (2011) explains how the composition of the top exporters is relevant in the transmission of international trade shocks. He states that the emergence of new big exporters might causes instability on the IFTN.

Players as Russia and Ukraine are more volatile due to export restrictions (mostly in 2006 and

2008), while Australia is more exposed to drought. However, we show that export crises might happen even in those countries that do not suffer from this kind of constraints. A signal of global trade expansion comes from the sheer number of active players in the market that for the exporters (importers) goes from 59 (154) in 1986 to 120 (180) in 2011.

From the demand side, the picture is more mixed and, in general, the market is less concentrated. The top 5 importers cover only a relatively small (and decreasing) shares of demand (about 25%), each one representing around 5% of global demand, in 2011. This also means that it is less probable that a drop in global export is induced by a fall in demand from a single country.

When looking at rice, maize, and soya (Fig B) we observe mixed results with the common feature of the markets - both from the demand and supply side - of being more concentrated than that of wheat. Maize has a demand structure dominated by Japan and China, which emerges only in very recent years, which cover together the 21% of the demand, that is moderately fragmented. The supply side, instead, is experiencing an openness to more competition, where more countries are participating to the IFTN. The glaring leader are the USA which exported about $55 \cdot 10^6$ tonnes (43.9%), followed by Argentina (14.4%) and Brazil (9.3%). Rice shows a demand structure close to that of wheat and highly fragmented.

The supply side (in 2011) is heavily dominated by the Asian exporters that account for the 77% of global trade, with the leadership of Thailand (38.4%), followed by Vietnam and India (14%), and Pakistan (11%). Finally, the global market of soya shows a demand heavily concentrated where the top 5 cover around 80% of the whole market. However, in 1986 the demand was more fragmented with a peak of 12% due to the Netherland, while in 2011 China Mainland alone covers almost the 67% of the demand. Even the supply side is still highly concentrated notwithstanding a descending trend toward more openness. The USA provided $61 \cdot 10^6$ tonnes in 2011, supplying more than half of the global market (52,3%), followed by Brazil (26,8%) and Argentina (8,3%).

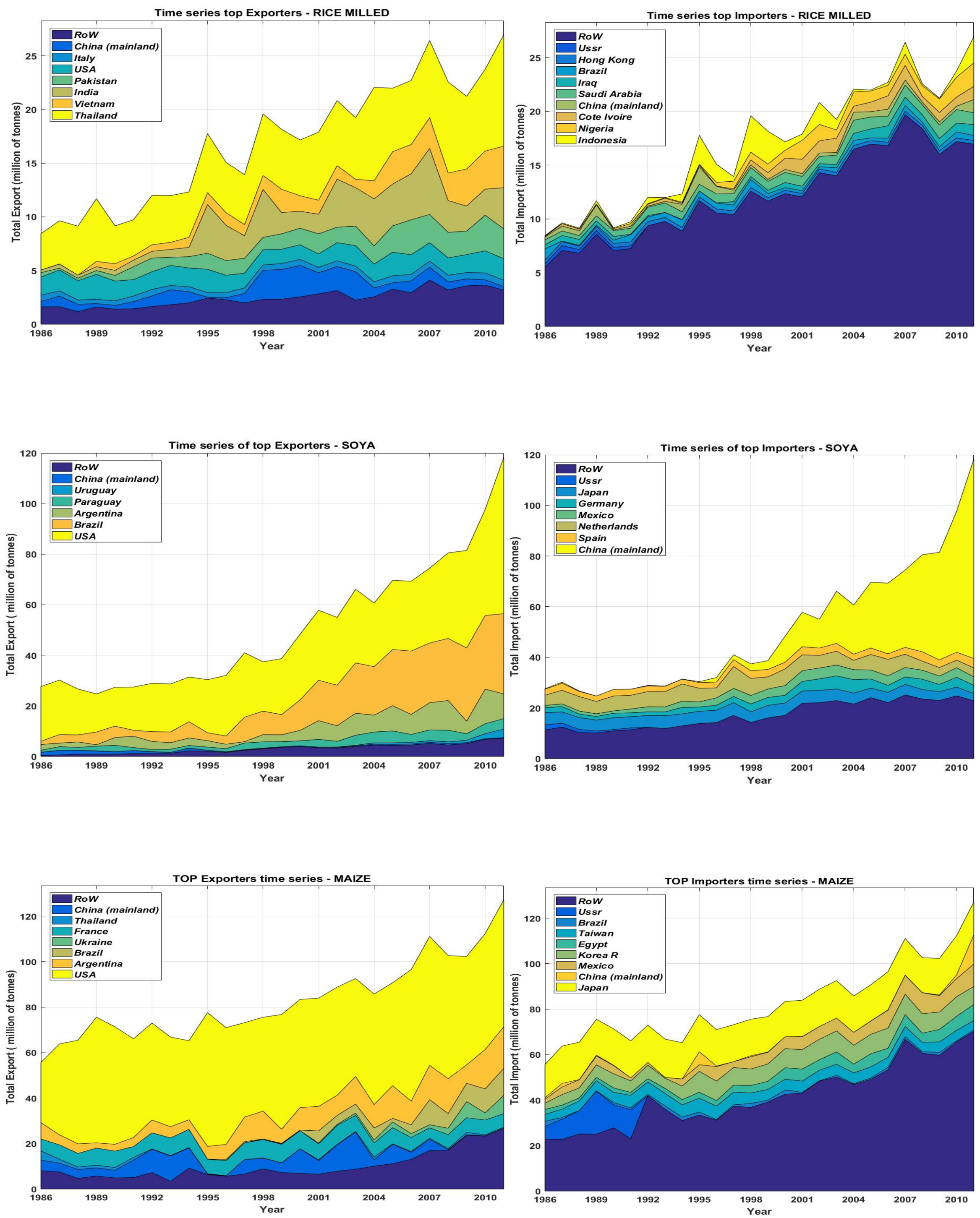

Fig B. Top exporters and importers over time. *Historical trend of the main export fluxes (left) and import fluxes (right), from 1986 to 2011, in million of tonnes. The Figure shows the time series of the exporters and importers which were among the top 5 in 1986 and/or in 2011 for rice (top), soya (center) and maize (bottom).*
