# Supplementary material for: Shock transmission in the International Food Trade Network
Source: PLoS One. 2018 Aug 8;13(8):e0200639. doi: 10.1371/journal.pone.0200639 (PMC6082532; doi:10.1371/journal.pone.0200639)
Supplement: S2 File — (PDF) [file pone.0200639.s002.pdf]

Supporting Information

S2 File. Country-scale Trade Shocks

Table A-C report the top 10 historical country-level negative food–supply shocks in the IFTN of rice, soya and maize from 1986 to 2011. It reports the volume and shares reduction, the years and duration of the crisis, and the effect on the ranking after the shock. The $\Theta_j(\tau)$ index indicates whether the shock propagates worldwide or whether the competitors offset the country-level trade shock.

Table A: Summary of the main indicators of the top 10 country-level crisis for rice.

rice	$\Delta E_j(\tau)$	**Years**	$\frac{\Delta E_j(\tau)}{E_j(t_0)}$ (%)	$\Delta E_j^w(\tau)$	$\Theta_j(\tau)$	Ranking $t_0 \rightarrow t_1$
India	-3.8	2007 - 2009	-62.1%	-5.2	-0.73	2 $\rightarrow$ 3
India	-2.9	1998 - 2000	-64.6%	-2.4	-1.19	2 $\rightarrow$ 3
Thailand	-2.8	2004 - 2005	-32.0%	-0.1	-37.29	1 $\rightarrow$ 1
Thailand	-2.5	1989 - 1991	-42.3%	-2.0	-1.26	1 $\rightarrow$ 1
India	-2.4	1995 - 1997	-53.8%	-3.8	-0.64	2 $\rightarrow$ 2
China	-2.1	2003 - 2005	-77.1%	2.8	0.75	3 $\rightarrow$8
Thailand	-1.7	2008 - 2009	-20.2%	-1.3	-1.28	1 $\rightarrow$ 1
Vietnam	-1.4	1999 - 2003	-63.7%	1.1	1.27	3 $\rightarrow$ 6
China	-1.3	1993 - 1995	-94.4%	5.8	0.23	3 $\rightarrow$ 15
Pakistan	-1.1	2006 - 2009	-32.8%	-1.5	-0.73	3 $\rightarrow$ 4

Table B: Summary of the main indicators of the top 10 country-level crisis for soya.

soya	$\Delta E_j(\tau)$	**Years**	$\frac{\Delta E_j(\tau)}{E_j(t_0)}$ (%)	$\Delta E_j^w(\tau)$	$\Theta_j(\tau)$	Ranking $t_0 \to t_1$
Argentina	-6.8	2008 - 2009	-57.9%	1.0	7.1	$3 \to 3$
USA	-6.6	1986 - 1989	-30.4%	-2.9	-2.3	$1 \to 1$
USA	-6.0	1997 - 1998	-23.5%	-3.6	-1.7	$1 \to 1$
USA	-4.0	2003 - 2004	-13.6%	-5.4	-0.7	$1 \to 1$
Argentina	-3.9	2010 - 2011	-28.4%	20.5	0.2	$3 \to 3$
Brazil	-3.3	1994 - 1995	-51.5%	-1.0	-3.2	$2 \to 2$
Brazil	-2.7	1989 - 1991	-54.1%	2.7	1.0	$2 \to 3$
Argentina	-2.4	1994 - 1997	-81.6%	9.6	0.3	$3 \to 6$
Argentina	-2.1	2005 - 2006	-20.9%	-0.3	-6.2	$3 \to 3$
Argentina	-2.1	2003 - 2004	-23.6%	-5.4	-0.4	$3 \to 3$

Table C: Summary of the main indicators of the top 10 country-level crisis for maize.

maize	$\Delta E_j(\tau)$	**Years**	$\frac{\Delta E_j(\tau)}{E_j(t_0)}$ (%)	$\Delta E_j^w(\tau)$	$\Theta_j(\tau)$	Ranking $t_0 \rightarrow t_1$
USA	-20.5	1989 1994	-37.1%	-10.3	-2.0	$1 \rightarrow 1$
USA	-17.6	1995 - 1998	-29.9%	-2.0	-8.6	$1 \rightarrow 1$
China	-13.7	2003 - 2004	-83.3%	-6.8	-2.0	$2 \rightarrow 5$
China	-10.9	1993 - 1996	-98.5%	4.1	2.6	$2 \rightarrow 15$
USA	-10.1	2006 - 2009	-17.5%	5.9	1.7	$1 \rightarrow 1$
Argentina	-7.3	2008 - 2009	-47.3%	-0.3	-23.1	$2 \rightarrow 2$
Argentina	-6.1	1998 - 1999	-49.7%	1.2	5.1	$2 \rightarrow 3$
China	-5.5	2005 - 2006	-64.0%	5.7	1.0	$3 \rightarrow 5$
Argentina	-4.9	1986 - 1989	-67.8%	19.9	0.2	$2 \rightarrow 4$
China	-4.8	2000 - 2001	-44.7%	0.5	9.1	$2 \rightarrow 4$
