# Supplementary material for: Shock transmission in the International Food Trade Network
Source: PLoS One. 2018 Aug 8;13(8):e0200639. doi: 10.1371/journal.pone.0200639 (PMC6082532; doi:10.1371/journal.pone.0200639)
Supplement: S3 File — (PDF) [file pone.0200639.s003.pdf]

Supporting Information

S3 File. Global-scale Trade Shocks

Here we report the same information contained in the Table 3 of Section 5 for rice, soya, and maize about the cross income area comparison for the top 5 worst global trade shocks. The ranking of the crises follows the magnitude of food loss in the IFTN. $\Delta F_k(\tau)$ and ΔE^w are expressed in million of tonnes, while $\frac{\Delta F_k(\tau)}{POP}$ in kg per capita. Price are deflated and computed following equation (4). The total percentage variation is computed as an average, based on the whole pre-crisis import level of each income group. The second row of total values corresponds to what we would obtain with the modified WB income thresholds (only in case of maize).

In the years 2010-11 the global average price of maize surged of almost +40% and E^w grew as well (+13%), while in the 1992-93 in view of a lack of global supply (-8% of E^w) the global price decreased of almost -5%. Soya exports rose of more than +8% in view of a global price spike of almost +50% in only one year (2007-08), while an export crisis, -9% from 1997-98, was not captured price dynamics (-19% in the same time span).

Table A: Cross Income Area comparison for the top 5 worst global trade shocks of rice in the period 1986-2011. *$\Delta F_k(\tau)$ and ΔE^w are expressed in million of tonnes, while $\frac{\Delta F_k(\tau)}{POP}$ in kg per capita. Price are deflated and computed following equation (4). The average percentage $\phi_k = \frac{\Delta F_k(\tau)}{F_k(t_0)}$ for each income group are in brackets.*

Global	$\Delta F_k(\phi_k)$				ΔE^w	$\frac{\Delta F_k(\tau)}{POP}$				**Price (ρ_t)**		
Crisis - rice	*Low*	*ML*	*UM*	*High*		*Low*	*ML*	*UM*	*High*	ρ_{t_0}	ρ_{t_1}	$\Delta\rho(\tau)$
I.(2007-2009)	-2.9 (-36%)	-2.3 (-27%)	0.2 (5%)	-0.2 (-2%)	-5.2	-1.17	-1.02	0.18	-0.15	386	622	+236
II.(1995-1997)	-2.1 (-36%)	-1.9 (-35%)	0.0 (1%)	0.1 (3%)	-3.8	-0.65	-2.14	0.06	0.12	306	366	+60
III.(1989-1990)	-2.3 (-55%)	-0.1 (-4%)	0.2 (13%)	-0.1 (-5%)	-2.4	-0.79	-0.16	0.32	-0.17	309	318	+9
IV.(1998-2000)	-0.8 (-13%)	-2.1 (-31%)	-0.4 (-13%)	0.9 (22%)	-2.4	-0.22	-2.34	-0.56	0.91	348	273	-75
V.(2002-2003)	0.0 (0%)	-2.3 (-39%)	0.4 (13%)	0.4 (8%)	-1.6	-0.01	-1.05	0.47	0.37	235	256	+21
TOT	-8.1 (-28%)	-8.7 (-27%)	0.3 (4%)	1.0 (5%)	-15.5	-2.85	-6.71	0.48	1.08			

Table B: Cross Income Area comparison for the top 5 worst global trade shocks of soya in the period 1986-2011. *$\Delta F_k(\tau)$ and ΔE^w are expressed in million of tonnes, while $\frac{\Delta F_k(\tau)}{POP}$ in kg per capita. Price are deflated and computed following equation (4). The average percentage $\phi_k = \frac{\Delta F_k(\tau)}{F_k(t_0)}$ for each income group are in brackets.*

Global	$\Delta F_k(\phi_k)$				ΔE^w	$\frac{\Delta F_k(\tau)}{POP}$				**Price** (ρ_t)		
Crisis - soya	*Low*	*ML*	*UM*	*High*		*Low*	*ML*	*UM*	*High*	ρ_{t_0}	ρ_{t_1}	$\Delta\rho(\tau)$
I.(1987-1989)	-0.4	-1.4	-0.7	-3.0	-5.5	-0.17	-1.39	-1.24	-3.60	185	240	+55
	(-95%)	(-43%)	(-18%)	(-13%)								
II.(2003-2004)	0.1	-1.2	-0.8	-3.6	-5.4	0.05	-0.52	-1.00	-3.63	227	276	+49
	(35%)	(-4%)	(-9%)	(-12%)								
III.(1997-1998)	0.9	0.1	-1.2	-3.4	-3.6	0.27	0.06	-2.15	-3.52	269	226	-43
	(44%)	(3%()	(-17%)	(-11%)								
IV.(2001-2002)	0.0	-1.9	0.3	-1.2	-2.8	0.02	-0.80	0.45	-1.26	175	193	+18
	(12%)	(-10%)	(4%)	(-4%)								
V.(1994-1995)	0.5	-0.5	-0.4	-0.6	-1.0	0.15	-0.62	-0.59	-0.66	212	210	-2
	(-28%)	(-8%)	(-2%)									
TOT	1.1	-4.8	-2.8	-11.9	-18.4	0.32	-3.27	-4.54	-12.66			
	(90%)	(-16%)	(-10%)	(-9%)								

Table C: Cross Income Area comparison for the top 5 worst global trade shocks of maize in the period 1986-2011. *$\Delta F_k(\tau)$ and ΔE^w are expressed in million of tonnes, while $\frac{\Delta F_k(\tau)}{POP}$ in kg per capita. Price are deflated and computed following equation (4). The average percentage $\phi_k = \frac{\Delta F_k(\tau)}{F_k(t_0)}$ for each income group are in brackets.*

Global	$\Delta F_k(\phi_k)$				ΔE^w	$\frac{\Delta F_k(\tau)}{POP}$				**Price** (ρ_t)		
Crisis - maize	*Low*	*ML*	*UM*	*High*		*Low*	*ML*	*UM*	*High*	ρ_{t_0}	ρ_{t_1}	$\Delta\rho(\tau)$
I.(1989-1991)	0.3	-5.2	-4.0	-0.6	-9.5	0.10	-5.90	-8.57	-0.65	125	123	-2
	(33%)	(-19%)	(-31%)	(-2%)								
II.(1992-1994)	3.0	0.0	-2.3	-9.5	-8.8	1.22	0.01	-2.62	-9.01	123	118	-5
	(126%)	(0%)	(-8%)	(-16%)								
III.(1995-1996)	-0.8	1.3	-7.9	-0.4	-7.7	-0.23	2.30	-11.92	-0.45	126	164	+38
	(-18%)	(12%)	(-48%)	(-1%)								
IV.(2002-2004)	-0.7	-1.2	-1.3	-3.6	-6.7	-0.28	-0.54	-1.65	-3.59	109	133	+24
	(-27%)	(-6%)	(-8%)	(-7%)								
V.(2007-2009)	-5.7	-0.9	2.6	-2.6	-6.6	-1.77	-1.02	3.77	-2.76	179	187	+8
	(-84%)	(-7%)	(22%)	(-6%)								
TOT	-3.8	-6.0	-12.9	-16.6	-39.4	-0.97	-5.14	-21.00	-16.45			
	(6.2%)	(-4%)	(-15%)	(-6.1%)								
TOT*	-3.8	-19.0	+0.1	-16.6	-39.4	-0.97	-18.70	+11.26	-16.45			
	(6.2%)	(-15.2%)	(+23%)	(-6.1%)								

Finally, we show the time series of price level and export normalised by mean (left) and scatter plot of their yearly percentage variations (right). Similarly to the case of wheat, the other staple foods show a weak tie between the dynamic of tonnes and price.

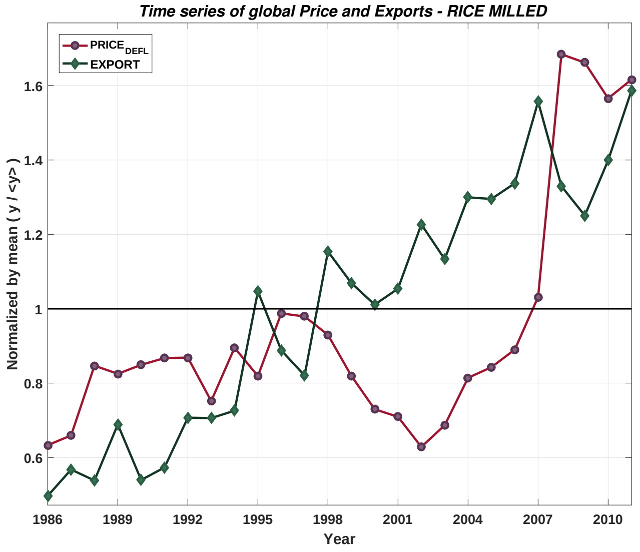

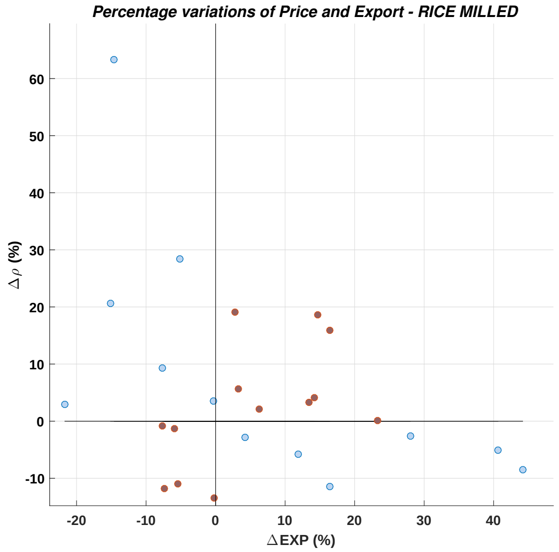

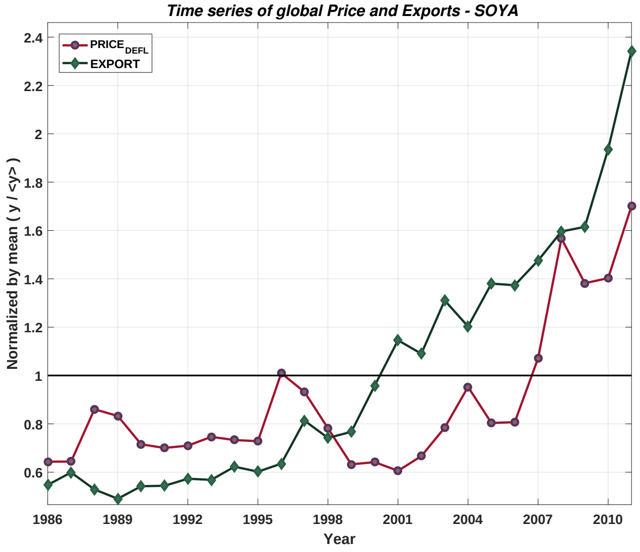

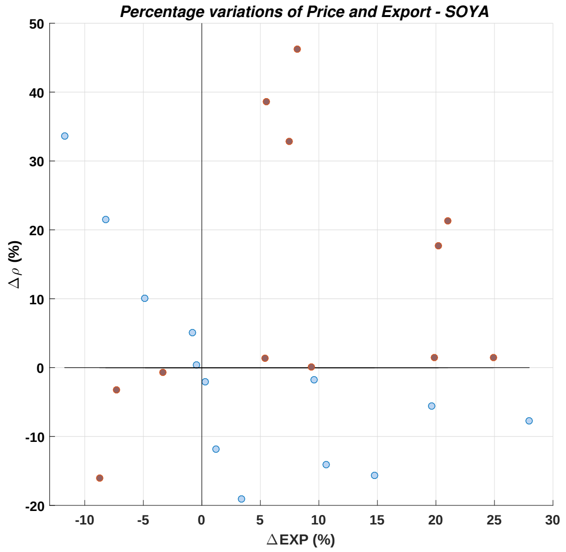

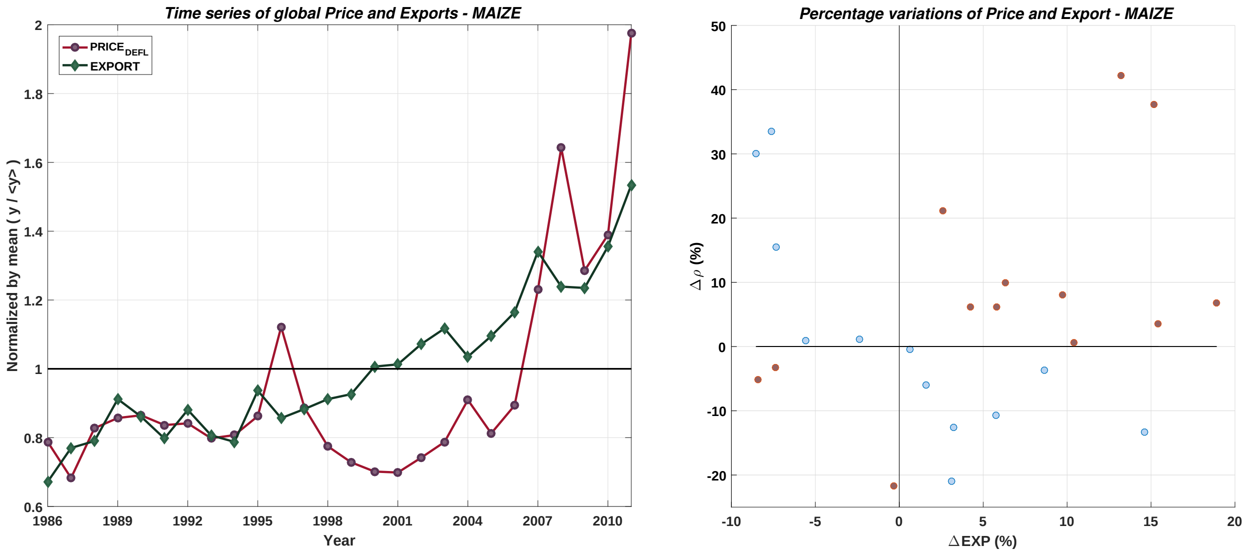

Fig A. Global export and price over time. *Left panel: Time series of average global price and global export, normalised by mean, for rice (top), soya (center) and maize (bottom). Right panel: scatter plot of yearly percentage variations of global price and export; red dots stand for positive correlations and blue dots for negative ones.*
